# Pragmatic Carbohydrate Quality Metrics in Relation to Glycemic Index, Glycemic Load, and Front-of-Pack Warning Labels in Grain Foods

**DOI:** 10.3390/foods13091299

**Published:** 2024-04-24

**Authors:** Mariane de Mello Fontanelli, Lais Duarte Batista, Angela Martinez-Arroyo, Dariush Mozaffarian, Renata Micha, Marcelo Macedo Rogero, Regina Mara Fisberg, Flavia Mori Sarti

**Affiliations:** 1 School of Arts, Sciences and Humanities (EACH), University of São Paulo, São Paulo 03828-000, SP, Brazil; marianefontanelli@usp.br; 2 School of Public Health, University of São Paulo, São Paulo 05508-030, SP, Brazil; laisduarte@usp.br (L.D.B.); mmrogero@usp.br (M.M.R.); rfisberg@usp.br (R.M.F.); 3 Food Behavior Research Center (CEIC), Faculty of Pharmacy, School Nutrition and Dietetics, University of Valparaíso, Valparaíso 2381850, Chile; angela.martinez@uv.cl; 4 Food is Medicine Institute, Friedman School of Nutrition Science and Policy, Tufts University, Boston, MA 02111, USA; dariush.mozaffarian@tufts.edu (D.M.); renata.micha@tufts.edu (R.M.); 5 Department of Food Science and Nutrition, University of Thessaly, 38334 Volos, Greece

**Keywords:** carbohydrate quality, dietary fiber, dietary sugars, glycemic index, glycemic load, warning labels

## Abstract

The challenges in the characterization of the nutritional quality of grain foods comprise obstacles to public health actions toward promotion of healthier grain-based foods. The present study investigated how carbohydrate metrics related to glycemic index (GI), glycemic load (GL), and warning labels of grain foods consumed by individuals living in São Paulo, Brazil. Information on intake of grain foods at individual level was obtained using 24 h recalls within a cross-sectional population-based survey conducted in 2015. There were 244 unique grain products reported by individuals in the survey, assessed through four metrics of carbohydrate quality, considering contents per 10 g of total carbohydrate: (1) ≥1 g fiber, (2) ≥1 g fiber and <1 g free sugars, (3) ≥1 g fiber and <2 g free sugars, and (4) ≥1 g fiber, and <2 g free sugars per 1 g of fiber. Outcomes included GI, GL, and inclusion of warning labels proposed by the Brazilian National Health Surveillance Agency (ANVISA), the Chilean Ministry of Health (1st and 3rd stages), and the Pan American Health Organization (PAHO). Metrics identified products with lower mean GI (−12.8 to −9.0 [*p*-values < 0.001]), and GL (−12.5 to −10.3 [*p*-values < 0.001]). Warning systems showed a certain degree of discrimination between products according to the metrics (*p*-value < 0.01 each); however, >50% of products with good nutritional quality according to the carbohydrate metrics still would receive warnings. Findings suggest that carbohydrate metrics identified products with lower GI and GL, and current warning labels may not adequately capture overall nutritional quality of grain foods.

## 1. Introduction

Grain foods are major energy sources in the global diet, presenting both potentially beneficial and adverse effects. A major part of dietary guidelines recommends higher consumption of whole grains and dietary fiber, as well as lower consumption of added sugars and refined grains, proposing dietary patterns protectively associated with cardiovascular disease and type 2 diabetes [1,2,3]. However, grain-based products usually comprise complex foods containing various combinations of whole/refined grains, dietary fibers, and added sugars, hindering efforts from consumers, industry, and policymakers to evaluate their overall healthfulness. In addition, the nutritional quality of carbohydrate-rich foods is further influenced by other partly overlapping but distinct traits, including glycemic index (GI), glycemic load (GL), and levels of vitamins and minerals [4,5,6]. The challenge of characterizing overall nutritional quality across all dimensions has hampered public health action, industry reformulation, consumer knowledge, and associated government policies toward healthier grain-based food options [7,8,9].

Recently, several simple metrics have been proposed and validated as practical measures of overall carbohydrate quality [7,10,11], aiming at incorporation of the balance between whole grains, bran, refined starch, and free sugars. The carbohydrate quality metrics have been based on the ratio of carbohydrate to fiber in whole wheat and recommendations for dietary fiber and added sugar intake from international agencies [12,13,14] and were shown to identify carbohydrate-providing foods with better nutritional profile, including higher contents of dietary fiber, protein, vitamins, and minerals, and lower contents of energy, sugar, and total and saturated fat. Higher consumption of foods containing at least 1 g of fiber per each 10 g of total carbohydrate (meeting the 10:1 metric) has been associated with lower levels of atherogenic dyslipidemia and insulin resistance [7,10,11,15].

Nevertheless, the relationship between carbohydrate quality metrics and other aspects of carbohydrate quality, e.g., GI and GL, remains unknown. The GI ranks foods containing carbohydrate according to their impact on blood glucose levels on a gram-to-gram basis, and the GL incorporates the amount consumed to the comparison between carbohydrate-providing foods [16]. Further validation of simple carbohydrate quality metrics in relation to GI and GL may support their adoption as pragmatic tools to support consumer decisions around carbohydrate-rich foods, especially due to the absence of widely available GI or GL values in retail settings [16]. Considering that postprandial glycemic response is a major link between carbohydrate intake and cardiometabolic diseases, the issue presents relevance in the public health perspective, especially due to the role of high-GL diets as key drivers of obesity and type 2 diabetes [17,18].

Furthermore, simple carbohydrate quality metrics could also support governments in decision-making processes toward health promotion policies, improving information available to guide consumer choices. Mandatory government front-of-pack (FOP) “black box” warning labels have been adopted to inform consumers in Chile, Brazil, Peru, Uruguay, and Mexico during the last decade [19,20]. However, FOP adopted in the countries focus on specific nutrients, e.g., sodium, fats, and sugars, lacking information on their interrelationships [19,21]. Therefore, several grain food products with diverse carbohydrate quality levels include FOP warnings [22], emphasizing the importance of understanding the relationship between previously validated metrics of carbohydrate quality and warning labels to identify whether FOP labels ensure consistency in public health communication regarding promotion of healthy diets. Thus, the present study investigated relationships between four pragmatic carbohydrate metrics, GI and GL, and FOP warning labels among grain-based foods consumed in São Paulo, Brazil. Based on the prior literature, we hypothesized that carbohydrate quality metrics would identify lower GI and GL options and foods less likely to present mandatory warning labels.

## 2. Materials and Methods

### 2.1. Population and Setting

This study was based on data from the Health Survey of São Paulo (Inquérito de Saúde de São Paulo), 3rd Edition, comprising a cross-sectional survey representative at population level conducted in the city of São Paulo, Brazil, in 2015 [23]. Located in the southeastern region of Brazil, the municipality is one of the largest cities in the Southern Hemisphere with a population of 11.5 million inhabitants in 2022 [24]. Participants were randomly selected through a two-stage sampling procedure: census tract (primary sampling unit), and households (secondary sampling unit). The sample size was planned to comprise 4250 participants, including a subsample of 900 individuals in three age groups (300 adolescents [12–19 years], 300 adults [20–59 years], and 300 older adults [≥60 years]) to obtain dietary information. The sample size allowed estimations of 0.50 proportions, with sampling error of 7% points, considering 95% confidence interval (CI) and design effect of 1.5 [25]. The final sample of the survey included 4058 participants, being 1741 individuals with at least one 24 h dietary recall included in the present analysis (Figure S1). Participants responding to the 24 h recalls presented similar characteristics to the overall original sample, except regarding age group, due to the higher proportion of adolescents and older adults in the sub-sample compared to the original sample: 23.2% vs. 13.5% were adolescents, and 22.7% vs. 16.1% were older adults in the subsample and original sample, respectively (Table S1).

### 2.2. Dietary Assessment

A total of 1741 participants completed one 24 h dietary recall in person, and 548 individuals completed the second recall through telephone interview [25]. Foods identified in the recalls were included in the product-specific analyses of this study. Dietary data were collected using the Multiple-Pass Method [26], and food items were converted into nutritional values using the Nutrition Data System for Research software version 2020 (NDSR, Nutrition Coordinating Center, University of Minnesota, Minneapolis, MN, USA). Nutrient contents of the food items were checked against the Brazilian National Food Composition Table [27], and values were corrected if the concordance rate was <80% or >120% compared to corresponding foods in the national table [25].

Grain foods were evaluated through disaggregation of dishes into ingredients using the United States Department of Agriculture (USDA) “grain products” food category [28]. In the present study, reported grain foods were assumed to be packaged (as opposed to obtained in restaurant or homemade) for comparison purposes, and therefore would be eligible to include FOP warning labels. Free sugar was estimated using the United Kingdom and Pan American Health Organization (PAHO) definitions, including all sugars naturally present in juiced or pureed fruit and vegetables, and sugars added by manufacturers, cooks, or consumers [29,30]. Acesulfame k, aspartame, saccharin, sucralose, isomaltose, maltitol, xylitol, mannitol, sorbitol, lactitol, or erythritol were defined as sweeteners based on the Brazilian legislation and availability in the NDSR software version 2020 [31].

### 2.3. Carbohydrate Metrics

Four previously validated carbohydrate metrics were evaluated in this study, each based on the contents of total carbohydrate, fiber, and/or free sugar of grain-based foods per 10 g of total carbohydrate [7,10,11]:

1. At least 1 g of fiber (10:1)—First proposed metric, recommended by the American Heart Association based on the naturally occurring carbohydrate-to-fiber ratio found in the whole wheat flour, and aiming to capture the relative balance between the contents of refined starch and added sugar vs. whole grain and bran [12,14].
2. At least 1 g of fiber and less than 1 g of free sugars (10:1:1)—Follows the same rationale of the 10:1 metric, with further incorporation of the recommendation of less than 5% of energy from free sugars [10,13,14]. According to the recommendations, a usual diet (e.g., 2000 kcal) should contain 50% of total calories from carbohydrates (e.g., 1000 kcal or 250 g), 25 g of dietary fiber, and less than 5% of total calories from free sugar (e.g., 100 kcal). Altogether, these recommendations are the 10:1:1 metric basis.
3. At least 1 g of fiber and less than 2 g of free sugars (10:1:2)—Follows the same rationale of the 10:1:1 metric, but allowing less than 10% of energy from free sugars [10,13,14].
4. At least 1 g of fiber, and less than 2 g of free sugars per each 1 g of fiber (10:1|1:2)—Follows the same rationale of the 10:1 and further limits the amount of free sugars depending on the fiber content [10,13,14]. This is particularly relevant to investigate whether the balance between fiber and free sugar would favorably affect cardiometabolic health considering differences in glycemic response.

### 2.4. Glycemic Index and Glycemic Load

The GI values of grain foods, using glucose as reference, were obtained from the NDSR software version 2020, which contains a compilation of data available in the literature. The GI information was included in the software using a similar methodology for adding GI values in the National Cancer Institute Diet History Questionnaire database [32]. Values were checked against information available in the Brazilian food composition table for consistency purposes [27]. Glycemic index values outside the 80–120% concordance rate were corrected using the Stata software (version 14.0, StataCorp LP, College Station, TX, USA). In addition to continuous analyses, grain-based foods were classified as low GI (GI ≤ 55), medium GI (GI = 56–69), or high GI (GI ≥ 70) [16].

We generated GL values of foods using the available carbohydrate mean per unique grain food as the amount of available carbohydrate in one food serving size, which was multiplied by the GI value and divided by 100. The available carbohydrate mean was estimated using all grain foods reported by study participants in the 24 h recalls. In addition to continuous assessment, the food items were categorized as low GL (GL ≤ 10), medium GL (GL = 11–19), or high GL (GL ≥ 20) [16].

### 2.5. Warning Label Systems

Grain foods were also categorized according to four FOP warning labels: the system adopted by the Brazilian National Health Surveillance Agency (Agência Nacional de Vigilância Sanitária—ANVISA), the systems adopted Chilean Ministry of Health (1st and 3rd stages), and the system proposed by the Pan American Health Organization (PAHO).

The ANVISA warning label in Brazil, implemented in October 2022, requires the presentation of FOP warnings in labels of foods containing (per 100 g) saturated fat ≥ 6 g, added sugar ≥ 15 g, or sodium ≥ 600 mg. Foods exempted of the Brazilian FOP warning labels include grains and flours without sugar, sodium, or saturated fat added to the product [33].

The Chilean FOP warning label system was implemented in stages, with the 1st stage adopted between June 2016 to June 2018, which labeled foods with added sugar, salt, or fat and containing (per 100 g) energy ≥ 350 kcal, saturated fat ≥ 6 g, total sugar ≥ 22.5 g, or sodium ≥ 800 mg [34,35]. The 3rd and last stage of the Chilean system was implemented in June 2019, labeling foods with added sugar, salt, or fat and containing (per 100 g) energy ≥ 275 kcal, saturated fat ≥ 4 g, total sugar ≥ 10 g, or sodium ≥ 400 mg [34,35].

The PAHO warning label system has been proposed as tool to support reduction in overweight and obesity prevalence, and aligns with the Brazilian Food-Based Dietary Guidelines [30,36], recommending labels for processed and ultra-processed foods according to the NOVA classification that also contain total fat ≥ 30% energy, saturated fat ≥ 10% energy, trans fat ≥ 1% energy, free sugar ≥ 10% energy, sodium ≥ 1 mg/1 kcal, or the presence of other sweeteners [30].

### 2.6. Statistical Analysis

Grain foods were assessed to identify adherence to the carbohydrate metrics, GI, and GL, and a requirement to include at least one criterion of the four FOP warning labels. The proportion of grain foods according to carbohydrate metrics was assessed, and kappa statistics were calculated according to food categories to assess agreement between metrics. The interpretation of agreement was based on none corresponding to values ≤ 0, slight corresponding to values between 0.01–0.20, fair corresponding to values between 0.21–0.40, moderate corresponding to values between 0.41–0.60, substantial corresponding to values between 0.61–0.80, and high corresponding to values between 0.81–1.00 [37].

Differences in mean GI and GL values of grain foods across carbohydrate metrics were assessed using univariate linear regressions with robust variance. In addition, univariate logistic regressions were estimated to verify differences in the probability of meeting each carbohydrate metric across categories of GI and GL (low, medium, and high), and probability of receiving at least one FOP warning label according to each carbohydrate metric.

Mean values of nutrients commonly considered in nutrient profiling models (i.e., energy [kcal], total sugar [g], added sugar [g], free sugar [g], total fat [g], saturated fat [g], trans fat [g], and sodium [mg]) were compared on a 100 g basis across carbohydrate metrics using univariate linear regression models with robust variance. The dependent variable referred to each nutrient being tested, and the independent variable referred to the dichotomous indicator of products meeting or not meeting each carbohydrate metric.

Analyses were performed according to food items to investigate carbohydrate quality metrics’ validity for assessing individual products. In secondary analyses, findings were weighted according to the frequency of consumption of foods (number of times the food item was reported in 24 h recalls) to assess metric performance at population level, which varied depending on the population dietary intake. Stata version 14.0 (StatCorp LLC, College Station, TX, USA) was used for statistical analyses and the survey module of the software was used for weighted analyses. A two-sided alpha < 0.05 was adopted.

## 3. Results

### 3.1. Grain Foods Meeting Each Carbohydrate Quality Metric

A total of 8511 grain foods were reported, including 244 unique items. The major parts of grain foods were “Cakes, cookies, pies, pastries, bars” (45.1%), followed by “Yeast bread, rolls” (20.5%), “Crackers and salty snacks from grain products” (11.1%), “Pastas, cooked cereals, rice” (9.8%), “Cereals, not cooked or NS as to cooked” (5.7%), “Flour and dry mixes” (3.7%), and “Quick breads” and “Pancakes, waffles, French toast, other grain products” (2.0% each) (Table 1).

The highest proportion of foods were those meeting the 10:1 (15.6%) criterion, followed by the 10:1|1:2 (13.1%), 10:1:2 (12.3%), and 10:1:1 (9.8%). The agreement between metrics was generally high (kappa = 0.86); however, it varied considerably across food categories (from kappa = −0.01 for “Cakes, cookies, pies, pastries, bars” to kappa = 0.94 for “Crackers and salty snacks from grain products”). Lower agreement was generally identified among food groups containing small number of unique food items. Findings weighted according to frequency of consumption are presented in Table S2.

### 3.2. Association of Carbohydrate Quality Metrics with Glycemic Index and Glycemic Load

The mean (SE) GI and GL of the 244 grain foods were 64.6 (0.7) and 19.9 (1.0), respectively (Table 2). Carbohydrate metrics identified products with lower mean GI and GL compared with products not meeting carbohydrate quality metrics. The highest GI difference was detected by the 10:1:1 criterion (−12.8, *p*-value < 0.001), followed by the 10:1|1:2 (−9.7, *p*-value < 0.001), the 10:1:2 (−9.2, *p*-value < 0.001), and the 10:1 (−9.0, *p*-value < 0.001). Results for the 10:1:2 metric were no longer significant after excluding foods with GI values ≤P1 and ≥P99 (Table S3). Regarding GL, the highest difference was identified through the 10:1:1 criterion (−12.5, *p*-value < 0.001), followed by the 10:1:2 (−10.9, *p*-value < 0.001), the 10:1|1:2 (−10.7, *p*-value < 0.001), and the 10:1 (−10.3, *p*-value < 0.001). Findings weighted according to the frequency of consumption are presented in Table S4.

**Figure 1:**
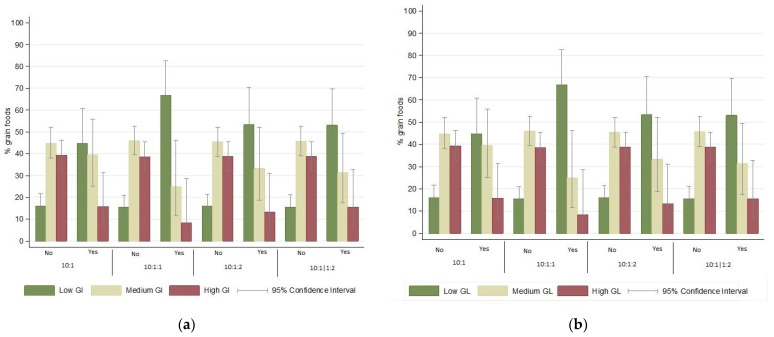
Proportion of food items according to categories of glycemic index (**a**) and glycemic load (**b**) in relation to their classification in carbohydrate quality metrics. GI, glycemic index; GL, glycemic load. Yes: Foods that met the respective carbohydrate metric. No: Foods that did not meet the respective carbohydrate metric. Metrics were applied per 10 g of total carbohydrate: (1) ≥1 g fiber (10:1), (2) ≥1 g fiber and <1 g free sugars (10:1:1), (3) ≥1 g fiber and <2 g free sugars (10:1:2), and (4) ≥1 g fiber, and <2 g free sugars per 1 g of fiber (10:1|1:2). Low GI ≤ 55, medium GI 56–69, high GI ≥ 70. Low GL ≤ 10, medium GL 11–19, high GL ≥ 20. Please see Table S4 for *p*-values obtained from logistic regression models.

The majority of grain foods had medium GI (70.9% [95% CI: 64.8–76.3]), followed by high GI (15.2% [95% CI: 11.2–20.3]) and low GI (13.9% [95% CI: 10.1–18.9]) (Table S5), Carbohydrate quality metrics identified food items more likely to be low GI than medium GI (*p*-values < 0.001 each), while the 10:1:1 also identified food items more likely to be low GI than high GI (*p*-value < 0.05). Nearly half of the foods meeting the metrics were low GI (ranging from 42.1% to 54.2% across metrics), compared to only one in ten foods not meeting the metrics (ranging from 9.0% to 10.8%). However, one in three foods meeting the metrics were high GI (ranging from 25.0% to 36.7%), compared with one in ten foods not meeting the metrics (ranging from 12.2% to 14.1%) (Figure 1). Findings weighted by frequency of consumption are presented in Table S5.

According to categories of GL, most grain foods had medium GL (43.9% [95% CI: 37.7–50.2]), followed by high GL (35.7% [95% CI: 29.9–41.9]) and low GL (20.5% [95% CI: 15.9–26.1]) (Table S5), and carbohydrate metrics identified food items more likely to be low GL than medium or high GL (*p*-values < 0.01 each). More than half of foods meeting the metrics were low GL (ranging from 44.7% to 66.7% across metrics), compared to one in five foods not meeting the metrics (ranging from 15.5% to 16%) (Figure 1). Findings weighted by frequency of consumption are presented in Table S5.

### 3.3. Association between Carbohydrate Quality Metrics and Warning Labels

**Figure 2:**
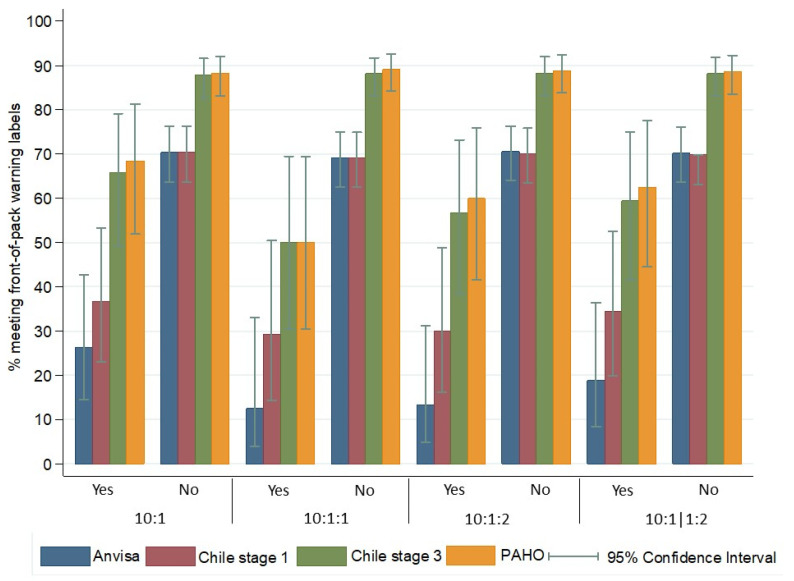
Proportion of grain foods meeting at least one criterion of front-of-pack warning label systems, according to their classification in carbohydrate quality metrics (*n* = 244). Metrics were applied per 10 g of total carbohydrate: (1) ≥1 g fiber (10:1), (2) ≥1 g fiber and <1 g free sugars (10:1:1), (3) ≥1 g fiber and <2 g free sugars (10:1:2), and (4) ≥1 g fiber, and <2 g free sugars per 1 g of fiber (10:1|1:2). The Brazilian National Health Surveillance Agency (Agência Nacional de Vigilância Sanitária—ANVISA) front-of-pack warning system tags foods with saturated fat ≥ 6 g/100 g, added sugar ≥ 15 g/100 g, or sodium ≥ 600 mg/100 g. Grains and flours without sugar, saturated fat, or sodium added to the product were not eligible for the warning label (*n* = 20). The 1st stage of the Chilean warning label tagged foods with energy ≥ 350 kcal/100 g, saturated fat ≥ 6 g/100 g, total sugar ≥ 22.5 g/100 g, or sodium ≥ 800 mg/100 g. In the 3rd stage, foods with energy ≥ 275 kcal/100 g, saturated fat ≥ 4 g/100 g, total sugar ≥ 10 g/100 g, or sodium ≥ 40 mg/100 g are tagged. Foods without sugar, sodium, or fat added to the product were not eligible for the Chilean warning label (*n* = 27). The Pan American Health Organization (PAHO) warning label tags processed and ultra-processed foods with total fat ≥ 30% food total energy (FTE), saturated fat ≥ 10% FTE, trans fat ≥ 1% FTE, free sugar ≥ 10% FTE, sodium ≥ 1 mg/1 kcal, or the presence of non-nutritive sweetener (*n* = 28). Yes: Foods that met the respective carbohydrate metric. No: Foods that did not meet the respective carbohydrate metric. All comparisons were statically significant (*p*-value < 0.001). Please see Table S5 for further details.

There was high proportion of grain products required to include at least one warning label of FOP systems considered in this study (Table S6), being 63.5% for Brazilian ANVISA (95% CI: 57.3–69.4), 65.2% for Chile 1st stage (95% CI: 58.9–70.9), 84.4% for Chile 3rd stage (95% CI: 79.3–88.5), and 85.2% for PAHO (95% CI: 80.2–89.2). Notably, the Brazilian ANVISA warning system and the 1st stage warning label of the Chilean system showed modest discrimination between products meeting or not meeting the carbohydrate quality metrics (Figure 2).

The ANVISA FOP system should require warning labels for 12.5–26.3% products meeting any of the carbohydrate quality metrics in Brazil, whilst 69.1–70.6% products not meeting metrics would be required to include warning labels. Considering the 1st stage of the Chilean FOP system, values ranged from 29.2–36.8% among products meeting any of the carbohydrate metrics and 69.1–70.4% among products not meeting the metrics.

The 3rd stage of the Chilean and the PAHO FOP systems showed slight discrimination of food products according to the carbohydrate quality metrics. More than half of the products meeting any of the four metrics still received a warning label according to the Chile 3rd stage (50.0–65.8%) and PAHO (50.0–68.4%) FOP systems, compared to 87.9–88.3% and 88.3–89.1% of products not meeting the metrics, respectively. Findings weighted by population frequency of consumption are detailed in Table S6.

### 3.4. Association between Carbohydrate Quality Metrics and Nutrients from the Warning Labels

All carbohydrate metrics identified products with lower content of total sugar (from −17 g for 10:1:1 to −11.4 g for 10:1), free sugar (from −16.3 g for 10:1:1 to −11.8 g for 10:1), added sugar (from −16.2 g for 10:1:1 to −11.7 g for 10:1), total fat (from −5.1 g for 10:1:2 to −3.4 g for 10:1), saturated fat (from −2.8 g for 10:1:2 and 10:1|1:2 to −2.1 g for 10:1), and trans fat (from −0.7 g for 10:1:1, 10:1:2, and 10:1|1:2 to −0.6 g for 10:1) per 100 g of each grain food.

There was a lack of significant differences for total energy or sodium contents (Table S7). The highest differences were identified by the 10:1:1 metric for total sugar (−17.0 g), free sugar (−16.3 g), and added sugar (−16.2 g); followed by the 10:1:2 metric (−15.3 g, −15.2 g, −15.2 g, respectively), the 10:1|1:2 (−14.3 g, −14.4 g, −14.4 g, respectively), and the 10:1 (−11.4 g, −11.8 g, −11.7 g, respectively).

The highest differences for total fat, saturated fat, and trans fat were identified by the 10:1:2 metric (−5.1 g, −2.8 g, −0.7 g, respectively), followed by the 10:1|1:2 metric (−4.8 g, −2.8 g, −0.7 g), the 10:1:1 metric (−4.4 g, −2.5 g, −0.7 g), and the 10:1 metric (−3.4 g, −2.1 g, −0.6 g). Although differences between metrics were relatively modest, the 10:1 metric was able to identify the highest proportion of food products (15.6%) (Table 1). In comparison, the 10:1:2 metric presented best performance considering the assessment of nutrients considered in this study, although it identified fewer food products (12.3%) (Table 1).

## 4. Discussion

The present study assessed the usefulness of carbohydrate quality metrics based on the balance between carbohydrate, fiber, and/or free sugar, related to GI and GL within food consumption patterns of the population of São Paulo municipality, one of the largest cities in South America. The four carbohydrate metrics investigated in this study presented similar performance in the identification of grain products according to the contents of major nutrients of concern and to the requirement for inclusion of front-of-pack (FOP) labels in diverse warning systems. The analyses performed using the four metrics showed their ability to identify food items with lower mean GI and GL, including weighted analyses based on frequency of consumption, although results were generally lacking statistically significance for GI.

The rationale for the carbohydrate quality metrics is to identify carbohydrate-providing foods with higher contents of whole grains and bran in comparison to foods with refined grains and added sugar. Previous studies showed that the carbohydrate quality metrics were able to identify foods with lower energy, sugar, and total and saturated fat, and higher content of dietary fiber, protein, vitamins, and minerals in investigations conducted in the United States, Australia, and Southeast Asian countries [7,10,11]. The findings of the present study regarding the possibility to identify foods with lower GI and GL further support the utility of the carbohydrate quality metrics in the selection of healthier grain foods.

The carbohydrate–insulin model of obesity places high GI diets as a major driver of the obesity pandemic, responsible for generating hormonal responses that increase fat deposition and produce positive energy balance [17]. Increased oxidative stress and related systemic low-grade inflammation are also adverse effects of high GI diets, contributing to the pathophysiology of insulin resistance and cardiometabolic diseases [38].

Higher postprandial glucose levels have been linked to hyperinsulinemia, metabolic inflammation, and dyslipidemia, as well as cardiometabolic diseases, including obesity, cardiovascular disease, and type 2 diabetes [38]. Although prediction of postprandial glycemic response is based on multiple factors (e.g., serum glycemic and lipid markers, dietary habits, anthropometrics, physical activity, genetics, and gut microbiota), practical approaches are required to support consumers’ decision-making processes from the public health perspective. Thus, signaling carbohydrate quality metrics may be adopted to reinforce health promotion strategies directed to reduction in cardiometabolic disease burden, especially in low- and middle-income countries marked by higher toll of disability-adjusted life years and mortality from cardiometabolic diseases [39,40,41].

A review investigating evidence of connections between foods, beverages, and nutrients in relation to cardiometabolic diseases showed results consistent with biological mechanisms, pointing to associations of GI and GL with coronary heart disease and type 2 diabetes (RR from 1.24 to 1.57) [42]. A review of randomized clinical trials indicated the effect of low GI/GL dietary patterns on cardiometabolic outcomes, presenting a high level of evidence in relation to glycated hemoglobin and high-density lipoprotein cholesterol, and a moderate level of evidence in relation to other health outcomes like body mass index, systolic and diastolic blood pressure, glycemia, and serum apolipoprotein B and C-reactive protein concentrations [43].

Despite the evidence on biological and pathophysiological mechanisms, the use of GI and GL to inform consumer choice and public policy remains challenging due to the absence of widely available GI and GL values for the major part of commonly consumed foods [16]. Although results were less consistent for GI, the findings of the present study suggest that the utilization of pragmatic metrics of carbohydrate quality, based on information readily available on food labels and local food composition tables, may help to guide consumers and policymakers towards promotion of lower GI/GL grain foods.

The carbohydrate quality metrics investigated in this study also allowed the identification of grain foods with favorable levels of certain nutrients of concern, e.g., total sugar, added sugar, free sugar, total fat, saturated fat, and trans fat, with similar performance. In addition, the comparative analyses of carbohydrate metrics against FOP warning labeling systems adopted in Latin American countries [19,20] showed that two systems presented modest performance in the identification of food items meeting the carbohydrate quality metrics: the Brazilian ANVISA and the 1st stage of the Chilean warning systems. Approximately 13–26% of products meeting the carbohydrate quality metrics would receive the Brazilian ANVISA warning label, and 29–37% would receive the Chilean 1st stage warning label, whereas ≥ 50% of products meeting the metrics should include warning label according to the Chile 3rd stage and the PAHO warning systems.

Indeed, there would be high proportion of grain products required to include FOP warning labels within certain food label systems investigated in the present study. Thus, considering the substantial amount of grain foods consumed daily in Latin American countries (mean intake of ~320 g/d) [44], the low sensitivity of certain warning label systems in the region could generate the misperception that the majority of grain products are equally unhealthy choices, hindering consumers’ decision-making processes towards healthier diets.

Given the successful identification of grain foods with lower mean GI/GL, total sugar, added sugar, free sugar, total fat, saturated fat, and trans fat through application of carbohydrate quality metrics, they appear to provide important discrimination between diverse grain products available for consumers, favoring the selection of grain foods that support reduction in exposure to cardiometabolic risk factors [15]. Therefore, the findings suggest that FOP warning systems implemented in Latin American countries should consider revisions of FOP labels to include one of the pragmatic carbohydrate quality metrics, which supported identification of grain foods with better carbohydrate quality than isolated nutrient thresholds.

Furthermore, three years after implementation of Chile’s FOP label warning system, there was decline in total sugar intake among children at school (−11.8 percentage points [pp]) and at home (−4.5 pp); however, studies also identified compensatory behavior in food consumption from other locations (e.g., restaurants, street, on transportation) with an increase of 5.8 pp [45]. High-sugar products comprised 39% of foods assessed pre-policy implementation against 30% post-policy implementation, indicating the replacement of sugar with non-nutritive sweeteners [46].

Overweight and obesity prevalence among children in Chile showed decreasing trends in the first-year post-policy implementation, followed by a rebound to previous levels in the following year, whilst overweight and obesity prevalence among adolescents increased from the implementation of the policy onwards [47]. The Chilean system only requires “high sugar” warning for products with sugar added into their formulation, i.e., influencing the free sugar component of the carbohydrate metrics. Thus, further investigations are required for assessment of potential cardiometabolic impacts of changes in the composition of foods and beverages after policy implementation, considering that the food industry may be compelled to modify food ingredients to minimize requirements of FOP labelling in its products.

The carbohydrate quality metrics were also previously compared to other food labeling systems (United Kingdom Office of Communications system, Food Standards Australia New Zealand, Singapore Healthier Choice Symbol, and Health Star Rating) in Australia, Malaysia, Philippines, Singapore, Thailand, and United States. The majority of foods meeting carbohydrate quality metrics were categorized into healthfulness thresholds for the nutrient profiling systems in comparison to foods not meeting carbohydrate quality metrics; however, proportions varied from approximately 70–85% versus 38–45% of products (respectively) in Australia and United States to 21–77% versus 1–20% (respectively) in Asian countries [10,11].

Public health communication based on clear and consistent messages is essential to improve dietary patterns and cardiometabolic health of populations. The information provided on food labels plays a central role in guiding the population toward healthier food choices [48]. Considering the importance of grain foods to global diets and their potential to contribute for the improvement of dietary quality, the concept of carbohydrate quality should be incorporated in food label warning systems for guidance in the selection of foods regarding certain nutrients of concern.

Although the information on contents of carbohydrate, fiber, and added sugar is mandatory on food labels in Brazil and other countries, using the carbohydrate quality metrics provides additional inputs for consumers’ decision-making processes, and trustworthy sources of data for estimation of carbohydrate quality metrics are required [33]. Therefore, further investigation on the metrics’ usefulness for FOP food labeling purposes is required considering that the findings indicate failures in the current warning label systems in capturing carbohydrate quality.

Additionally, the similarities identified in the performance of metrics investigated in the present study show that the proportion of food items meeting the criteria may be important to choose the metrics for FOP labels [10,11]. In this sense, the 10:1 comprises the metric based on straightforward method for calculation and interpretation, being also comprehensive in capturing nutritional quality of foods. However, FOP warning systems focusing on decrease in consumption of added sugars may prioritize other metrics (10:1:1, 10:1:2, or 10:1|1:2), although evidence has shown similar metabolic and health impacts of added sugar and refined starch [49,50].

It is worth mentioning that the carbohydrate quality metrics equally consider naturally occurring fiber and extracted synthetic fiber added to foods [3]. The WHO recommendation on carbohydrate intake covers naturally occurring fiber and highlights the need to further investigate disease outcomes associated with extracted or synthetic fiber before including in the recommendation [3].

Potential limitations of the present study should be acknowledged. First, datasets on nutritional composition of foods available in Brazil lacks information on GI, and the assessment of GI was available for few locally consumed food items (n = 52) [27]. The GI values in the NDSR software version 2020 were compared to information available in the Brazilian Food Composition Table to ascertain the conformity of GI between sources of information.

Second, the warning label systems were selected to evaluate grain foods since they were recently adopted in Brazil and other Latin American countries [19]; therefore, the interpretation of the results are restricted to the four warning label systems tested, and may lack generalizability to other systems. However, findings may be similar to other FOP warning systems that focus solely on food ingredients or nutrients linked to negative health outcomes (i.e., obesity, chronic diseases, among others).

Third, the present study focused on foods reported by 1741 adolescents and adults living in São Paulo, Brazil. While the survey sample was designed to be representative at population level, additional studies encompassing a comprehensive range of grain foods might be captured in larger samples or surveys conducted in other parts of Brazil or other countries. The findings support evidence from previous studies in high-income countries [10,11]; however, further investigation should be performed in other low- and middle-income countries, especially considering the role of educational attainment and food literacy in adherence to label warnings [51,52,53]. In addition, the low number of foods that met the carbohydrate metrics may explain some of the observed inconsistencies for GI results, and larger sample sizes are required to further investigate this topic.

Finally, the strengths of this study refer to the evaluation of grain foods according to food items consumed by the population within the largest city of South America, including analyses weighted by frequency of consumption [24]. To our knowledge, this is the first study comparing the carbohydrate metrics to GI and GL. The findings also provide evidence on the carbohydrate metrics in relation to FOP warning labels adopted by middle-income countries in Latin America, where a substantial amount of grain foods is consumed and a high burden of cardiometabolic diseases occurs [41,44].

## 5. Conclusions

The four carbohydrate quality metrics investigated showed high accuracy in the identification of grain foods with lower GI and GL, and certain nutrients of concern within current international recommendations for healthy diets. The findings highlight the potential role of pragmatic carbohydrate quality metrics within FOP warning systems, being supplementary tools for decision-making processes of consumers, industry, and policymakers to shift dietary patterns toward healthier grain foods. FOP warning systems in Latin America showed certain degree of discrimination between grain foods with higher or lower carbohydrate quality within the criteria of the four metrics; however, a substantial proportion of products meeting the metrics would receive FOP warning labels according to the systems implemented in Chile (3rd stage) and proposed by the PAHO. Our analysis suggests that the current FOP food label systems may be inadequate to capture the overall nutrition quality of grain foods, particularly regarding carbohydrate quality.

## Figures and Tables

**Table 1:** Grain foods meeting the carbohydrate metrics consumed by the population aged ≥12 years in São Paulo, 2015 Health Survey of São Paulo.

Grain Food Categories ^1^	# ^2^	% (95% CI)	Percent Meeting Each Metric ^3^				Kappa
			10:1	10:1:1	10:1:2	10:1|1:2	
			% (95%CI)	% (95%CI)	% (95%CI)	% (95%CI)	
Flour and dry mixes	9	3.8 (1.9–7.0)	55.6 (23.5–83.5)	55.6 (23.5–83.5)	55.6 (23.5–83.5)	55.6 (23.5–83.5)	1.00
Yeast breads, rolls	50	20.5 (15.9–26.1)	18.0 (9.5–31.3)	10.0 (4.2–22.1)	18.0 (9.5–31.3)	18.0 (9.5–31.3)	0.85
Quick breads	5	2.0 (0.9–4.9)	0.0 (-)	0.0 (-)	0.0 (-)	0.0 (-)	0.00
Cakes, cookies, pies, pastries, bars	110	45.1 (38.9–51.4)	4.5 (1.9–10.6)	0.0 (-)	0.0 (-)	0.0 (-)	−0.01
Crackers and salty snacks from grain products	27	11.1 (7.7–15.7)	18.5 (7.8–38.1)	14.8 (5.5–34.0)	18.5 (7.8–38.1)	18.5 (7.7–38.1)	0.94
Pancakes, waffles, French toast, other grain products	5	2.0 (0.9–4.9)	0.0 (-)	0.0 (-)	0.0 (-)	0.0 (-)	0.00
Pastas, cooked cereals, rice	24	9.8 (6.7–14.3)	25.0 (11.4–46.3)	25.0 (11.4–46.2)	25.0 (11.4–46.2)	25.0 (11.4–46.2)	1.00
Cereals, not cooked or NS as to cooked	14	5.7 (3.4–9.5)	57.1 (30.7–80.1)	28.6 (10.7–57.3)	35.7 (15.1–63.5)	50.0 (25.1–74.9)	0.66
Overall	244	100.0 (-)	15.6 (11.5–20.7)	9.8 (6.7–14.3)	12.3 (8.7–17.1)	13.1 (9.4–18.0)	0.86

CI, confidence interval; NS, not specified. ^1^ Food groups “Grain mixtures, frozen plate meals, soups” and “Meat substitutes, mainly cereal protein” were suppressed given the lack of products within the categories. ^2^ Absolute number of grain products in the 2015 Health Survey of São Paulo dataset. ^3^ Metrics were applied per 10 g of total carbohydrate: (1) ≥1 g fiber (10:1), (2) ≥1 g fiber and <1 g free sugars (10:1:1), (3) ≥1 g fiber and <2 g free sugars (10:1:2), and (4) ≥1 g fiber, and <2 g free sugars per 1 g of fiber (10:1|1:2).

**Table 2:** Glycemic index and glycemic load of grain foods according to categorization in carbohydrate quality metrics, 2015 Health Survey of São Paulo.

Carbohydrate Metrics ^1^	Glycemic Index				Glycemic Load			
	Mean (SE)	Median (IQR)	Diff. (SE) ^2^	*p*-Value	Mean (SE)	Median (IQR)	Diff. (SE)	*p*-Value
10:1								
Yes ^3^	57.0 (2.5)	61.4 (25.0)	−9.0 (1.9)	<0.001	11.2 (1.2)	10.9 (10.5)	−10.3 (1.7)	<0.001
No ^4^	66.0 (0.7)	66.8 (4.3)			21.5 (1.2)	17.4 (14.7)		
10:1:1								
Yes	53.0 (3.5)	50.8 (30.4)	−12.8 (2.2)	<0.001	8.6 (1.5)	7.7 (9.6)	−12.5 (1.8)	<0.001
No	65.8 (0.6)	66.7 (4.6)			21.1 (1.1)	17.1 (13.9)		
10:1:2								
Yes	56.5 (3.1)	62.9 (30.0)	−9.2 (2.1)	<0.001	10.3 (1.4)	9.1 (9.6)	−10.9 (1.8)	<0.001
No	65.7 (0.7)	66.6 (4.4)			21.2 (1.1)	17.1 (14.2)		
10:1|1:2								
Yes	56.2 (3.0)	57.8 (28.4)	−9.7 (2.0)	<0.001	10.6 (1.4)	9.1 (10.9)	−10.7 (1.8)	<0.001
No	65.8 (0.7)	66.7 (4.4)			21.3 (1.1)	17.1 (14.2)		
Overall	64.6 (0.7)	66.6 (5.4)			19.9 (1.0)	16.1 (14.1)		

IQR, interquartile range; SE, standard error; Diff., difference. ^1^ Metrics were applied per 10 g of total carbohydrate: (1) ≥1 g fiber (10:1), (2) ≥1 g fiber and <1 g free sugars (10:1:1), (3) ≥1 g fiber and <2 g free sugars (10:1:2), and (4) ≥1 g fiber, and <2 g free sugars per 1 g of fiber (10:1|1:2). ^2^ β coefficient for linear regression model with robust variance. ^3^ Yes: Foods that met the respective carbohydrate metric. ^4^ No: Foods that did not meet the respective carbohydrate metric.

## Data Availability

The original contributions presented in the study are included in the article/Supplementary Materials, further inquiries can be directed to the corresponding author.

## Supplementary Materials

The following supporting information can be downloaded at: https://www.mdpi.com/article/10.3390/foods13091299/s1. Table S1. Characteristics of participants in the 2015 Health Survey of Sao Paulo, according to dietary recall information; Table S2. Grain foods meeting carbohydrate metrics weighted by frequency of consumption by the population of Sao Paulo ≥12 years, 2015 Health Survey of Sao Paulo; Table S3. Glycemic index and glycemic load of grain foods according to categorization in carbohydrate quality metrics excluding foods with GI values ≤P1 and ≥P99 (*n* = 7), 2015 Health Survey of Sao Paulo; Table S4. Comparison of glycemic index and glycemic load of grain foods meeting or not meeting each carbohydrate metric weighted by frequency of consumption, 2015 Health Survey of Sao Paulo; Table S5. Comparison of glycemic index and glycemic load of grain foods according to the carbohydrate metrics, 2015 Health Survey of Sao Paulo; Table S6. Comparison of consumed grain foods meeting at least one criterion of the front-of-pack warning labels according to carbohydrate metrics, 2015 Health Survey of Sao Paulo (*n* = 244); Table S7. Contents of critical nutrients in nutrient profiling models according to carbohydrate quality metrics, 2015 Health Survey of Sao Paulo; Figure S1. Flowchart of study participants, 2015 Health Survey of Sao Paulo.
