# Supplementary material for: Pragmatic Carbohydrate Quality Metrics in Relation to Glycemic Index, Glycemic Load, and Front-of-Pack Warning Labels in Grain Foods
Source: Foods. 2024 Apr 24;13(9):1299. doi: 10.3390/foods13091299 (PMC11083290; doi:10.3390/foods13091299)

Supplementary Material - Foods

Pragmatic carbohydrate quality metrics in relation to glycemic index, glycemic load, and front-of-pack warning labels in grain foods

Mariane de Mello Fontanelli, Lais Duarte Batista, Angela Martinez Arroyo, Dariush Mozaffarian, Renata Micha, Marcelo Macedo Rogero, Regina Mara Fisberg, Flavia Mori Sarti

Corresponding author: Flavia Mori Sarti, School of Arts, Sciences and Humanities (EACH), University of São Paulo, São Paulo-SP, Brazil

E-mail: flamori@usp.br

Table of contents

Table S1. Characteristics of participants in the 2015 Health Survey of Sao Paulo, according to dietary recall information. 2

Table S2. Grain foods meeting carbohydrate metrics weighted by frequency of consumption by the population of Sao Paulo ≥12 years, 2015 Health Survey of Sao Paulo. 3

Table S3. Glycemic index and glycemic load of grain foods according to categorization in carbohydrate quality metrics excluding foods with GI values ≤P1 and ≥P99 (n=7), 2015 Health Survey of Sao Paulo. 4

Table S4. Comparison of glycemic index and glycemic load of grain foods meeting or not meeting each carbohydrate metric weighted by frequency of consumption, 2015 Health Survey of Sao Paulo. 5

Table S5. Comparison of glycemic index and glycemic load of grain foods according to the carbohydrate metrics, 2015 Health Survey of Sao Paulo. 6

Table S6. Comparison of consumed grain foods meeting at least one criterion of the front-of-pack warning labels according to carbohydrate metrics, 2015 Health Survey of Sao Paulo (n=244). 8

Table S7. Contents of critical nutrients in nutrient profiling models according to carbohydrate quality metrics, 2015 Health Survey of Sao Paulo. 10

Figure S1. Flowchart of study participants, 2015 Health Survey of Sao Paulo. 11

Table S1. Characteristics of participants in the 2015 Health Survey of Sao Paulo, according to dietary recall information.

Population characteristics	Sample (n=4,058)		Subsample with at least one dietary recall (n=1,741) [e]	
	n	% (95% CI)[c]	n	% (95% CI)
Age group, years				
12-19	864	13.5 (12.4-14.7)	553	23.2 (20.9-25.7)
20-59	2,169	70.4 (68.71-72.1)	643	54.2 (51.5-56.8)
≥ 60	1,025	16.1 (14.3-17.9)	545	22.7 (19.9-25.7)
Sex				
Male	1,774	46.9 (45.3-48.4)	837	50.0 (47.2-52.9)
Female	2,284	53.1 (51.6-54.7)	904	50.0 (47.1-52.8)
Education level [a]				
< High school graduate	2,088	44.9 (41.7-48.2)	980	50.8 (47.0-54.7)
High school graduate/equivalent	956	25.2 (23.4-27.1)	366	22.4 (20.1-24.9)
College or some college	931	29.9 (26.2-33.8)	358	26.8 (23.1-30.8)
Family income per capita [a,b]				
≤ 1 minimum wage	1,722	40.9 (36.9-45.1)	769	42.6 (37.8-47.6)
> 1 and ≤ 3 minimum wages	1,227	33.2 (30.0-36.5)	517	32.9 (29.0-37.1)
> 3 minimum wages	261	8.8 (6.9-11.3)	123	9.6 (7.0-13.0)
Don't know response	643	17.1 (13.1-22.0)	257	14.9 (10.6-20.6)
Ethnicity [a]				
White and yellow	2,066	52.9 (49.5-56.3)	865	51.1 (47.4-54.8)
Black, brown, and indigenous	1,963	47.1 (43.7-50.5)	863	48.9 (45.2-52.7)

CI, confidence interval.
[a] Numbers may not sum to the total number of participants due to missing data.
[b] Minimum wage was 236.17 US dollars (788 Brazilian Real) in 2015.
[c] Percentages and 95% confidence intervals were survey weighted.

Table S2. Grain foods meeting carbohydrate metrics weighted by frequency of consumption by the population of Sao Paulo ≥12 years, 2015 Health Survey of Sao Paulo.

Grain food categories [a]	# [b]	% (95%CI) [c]	Percent meeting each metric [d]			
			10:1	10:1:1	10:1:2	10:1|1:2
			% (95%CI)	% (95%CI)	% (95%CI)	% (95%CI)
Flour and dry mixes	9	10.0 (1.3-0.5)	1.4 (0.1-11.8)	1.4 (0.1-11.8)	1.4 (0.1-11.8)	1.4 (0.1-11.8)
Yeast breads, rolls	50	30.3 (08.3-67.5)	6.1 (0.9-32.1)	0.8 (0.2-4.2)	6.1 (0.9-32.1)	6.1 (0.9-32.1)
Quick breads	5	0.5 (0.1-3.1)	0.0 (-)	0.0 (-)	0.0 (-)	0.0 (-)
Cakes, cookies, pies, pastries, bars	110	7.7 (3.2-17.4)	2.3 (0.7-7.1)	0.0 (-)	0.0 (-)	0.0 (-)
Crackers and salty snacks from grain products	27	7.1 (1.9-23.1)	6.4 (1.19-27.9)	6.2 (1.1-27.7)	6.4 (1.2-27.9)	6.4 (1.2-27.9)
Pancakes, waffles, French toast, other grain products	5	0.2 (00.0-1.07)	0.0 (-)	0.0 (-)	0.0 (-)	0.0 (-)
Pastas, cooked cereals, rice	24	42.4 (12.4-79.2)	0.9 (0.1-7.2)	0.9 (0.1-7.2)	0.9 (0.1-7.2)	0.9 (0.1-7.2)
Cereals, not cooked or NS as to cooked	14	1.8 (0.5-5.8)	69.2 (26.7-93.3)	43.8 (9.1-85.9)	44.8 (9.6-86.1)	51.3 (13.5-87.7)
**Overall**	244	100.0 (-)	4.3 (1.5-11.7)	2.0 (0.7-6.0)	3.6 (1.2-10.9)	3.8 (1.2-11.0)

CI, confidence interval.

[a] Food groups “Grain mixtures, frozen plate meals, soups” and “Meat substitutes, mainly cereal protein” were suppressed given the lack of products meeting these categories, since grain foods were evaluated with mixed dishes disaggregated.

[b] Absolute number of grain products in the 2015 Health Survey of São Paulo dataset.

[c] Products were weighted by their actual reported consumption levels (products consumed more frequently received larger weight).

[d] Metrics were applied per 10g of total carbohydrate: 1) ≥1g fiber (10:1), 2) ≥1g fiber and <1g free sugars (10:1:1), 3) ≥1g fiber and <2g free sugars (10:1:2); and 4) ≥1g fiber and, per 1 g of fiber, < 2g free sugars (10:1|1:2).

The most commonly consumed food groups weighted by the frequency of consumption in the population were “Pastas, cooked cereals, rice” (42.4%), followed by “Yeast bread, rolls” (30.3%), “Flour and dry mixes” (10%), “Cakes, cookies, pies, pastries, bars” (7.7%), “Crackers and salty snacks from grain products” (7.1%), “Cereals, not cooked or NS as to cooked” (1.8%), “Quick breads” (0.5%), and “Pancakes, waffles, French toast, other grain products” (0.2%). Notably, when weighted by frequency of consumption, the proportion of grain foods meeting each metric was much lower than the analysis by food item. The 10:1 metric was met by 4.3% of grain foods, followed by the 10:1|1:2 (3.8%), 10:1:2 (3.6%) and 10:1:1 (2%), indicating that healthier grain foods meeting the metrics were less frequently consumed by the population than products that did not meet the metrics.

Table S3. Glycemic index and glycemic load of grain foods according to categorization in carbohydrate quality metrics excluding foods with GI values ≤P1 and ≥P99 (n=7), 2015 Health Survey of Sao Paulo.

**Carbohydrate metrics [1]**	**Glycemic index**				**Glycemic load**			
	**Mean (SE)**	**Median (IQR)**	**Diff. (SE) [2]**	**P-value**	**Mean (SE)**	**Median (IQR)**	**Diff. (SE) [b]**	**P-value**
10:1								
Yes	59.8 (2.1)	64.9 (23.5)	-5.8 (2.2)	0.008	11.9 (1.2)	11.8 (9.5)	-9.7 (1.7)	<0.001
No	65.6 (0.6)	66.7 (4.3)			21.6 (1.2)	17.6 (14.9)		
10:1:1								
Yes	57.1 (3.1)	56.9 (26.9)	-8.4 (3.1)	0.007	9.5 (1.6)	8.6 (8.2)	-11.8 (1.9)	<0.001
No	65.5 (0.6)	66.7 (4.4)			21.2 (1.1)	17.1 (13.8)		
10:1:2								
Yes	60.1 (2.7)	66.6 (4.3)	-5.3 (2.7)	0.051	11.2 (1.5)	10.7 (11.1)	-10.1 (1.8)	<0.001
No	65.3 (0.6)	67.9 (25.1)			21.4 (1.1)	17.1 (14.1)		
10:1|1:2								
Yes	59.4 (2.5)	67.1 (24.4)	-6.1 (2.5)	<0.001	11.4 (1.5)	10.7 (11.1)	-10.0 (1.8)	<0.001
No	65.5 (0.6)	66.6 (4.3)			21.4 (1.1)	17.1 (14.11336)		
**Overall**	64.7 (0.6)	66.7 (5.1)			20.2 (1.0)	16.3 (14.0)		

IQR, interquartile range; SE, standard error; Diff., difference.
[1] Metrics were applied per 10g of total carbohydrate: 1) ≥1g fiber (10:1), 2) ≥1g fiber and <1g free sugars (10:1:1), 3) ≥1g fiber and <2g free sugars (10:1:2); and 4) ≥1g fiber and, per 1 g of fiber, < 2g free sugars (10:1|1:2).
[2] β coefficient for linear regression model with robust variance.
[3] Yes, foods that met the respective carbohydrate metric.
[4] No, foods that did not meet the respective carbohydrate metric.

Table S4. Comparison of glycemic index and glycemic load of grain foods meeting or not meeting each carbohydrate metric weighted by frequency of consumption, 2015 Health Survey of Sao Paulo.

**Carbohydrate metrics** [1]	**Glycemic index**				**Glycemic load**			
	**Mean (SE)**	**Median (IQR)**	**Diff. (SE)** [2]	**P-value** [3]	**Mean (SE)**	**Median (IQR)**	**Diff. (SE)**	**P-value**
10:1								
Yes [4]	58.6 (6.8)	67.9 (24.4)	-7.1 (7.2)	0.33	7.9 (2.4)	11.1 (10.8)	-10.7 (2.8)	**<0.001**
No [5]	65.7 (2.3)	68.4 (10.2)			21.4 (2.3)	18.3 (11.5)		
10:1:1								
Yes	47.1 (7.8)	47.6 (39.9)	-18.7 (8.1)	**0.02**	7.9 (2.4)	4.5 (8.4)	-13.4 (3.3)	**<0.001**
No	65.8 (2.2)	69 (10.2)			21.4 (2.3)	18.3 (11.5)		
10:1:2								
Yes	58.2 (8.0)	72 (30.1)	-7.5 (8.3)	0.37	10.0 (1.7)	11.1 (6.6)	-11.5 (2.8)	**<0.001**
No	65.7 (2.3)	68.3 (10.2)			21.2 (4.4)	19.9 (22.7)		
10:1|1:2								
Yes	58.0 (7.8)	72 (26.91)	-7.7 (8.1)	0.34	10.4 (1.7)	11.1 (7.7)	-11.1 (2.8)	**<0.001**
No	65.7 (2.3)	68.3 (10.2)			21.5 (2.3)	18.3 (11.5)		
**Overall**	65.4 (9.1)	68.4 (10.2)			21.1 (2.3)	18.3 (11.5)		

Diff., difference; IQR, interquartile range; SE, standard error.
[1] Metrics were applied per 10g of total carbohydrate: 1) ≥1g fiber (10:1), 2) ≥1g fiber and <1g free sugars (10:1:1), 3) ≥1g fiber and <2g free sugars (10:1:2); and 4) ≥1g fiber and, per 1 g of fiber, < 2g free sugars (10:1|1:2).
[2] β coefficient for linear regression weighted by frequency of consumption.
[3] P-values marked with bold indicate statistically significant p-values.
[4] Yes, foods that met the respective carbohydrate metric.
[5] No, foods that did not meet the respective carbo-hydrate metric.

Weighted by the population frequency of consumption, the mean GI was similar to the non-weighted value (65.4 *versus* 64.6, respectively), but the mean GL increased from 19.9 g in the non-weighted GL to 21.1 g in the weighted analysis, indicating that food items with higher GL were more frequently consumed. The difference between GI across carbohydrate metrics was no longer significant, except for the 10:1:1, which detected products with lower GI (-18.7, P value=0.022) when compared to those not meeting this metric.

All carbohydrate metrics identified grain foods with lower GL when the consumption of São Paulo's population was considered. The mean GL difference between grain foods meeting the carbohydrate metrics and not meeting the metrics was highest for the 10:1:1 (-13.4 g, P value<0.001) followed by the 10:1:2 (- 11.5 g, P value<0.001), the 10:1|1:2 (- 11.1 g, P value<0.001), and the 10:1 (-10.7 g, P value<0.001).

Table S5. Comparison of glycemic index and glycemic load of grain foods according to the carbohydrate metrics, 2015 Health Survey of Sao Paulo.

Indicators	10:1 [3]				10:1:1				10:1:2				10:1|1:2				Overall
	Yes [4] % (95% CI)	No [5] % (95% CI)	OR (95% CI)	P-value	Yes % (95% CI)	No % (95% CI)	OR (95% CI)	P-value	Yes % (95% CI)	No % (95% CI)	OR (95% CI)	P-value	Yes % (95% CI)	No % (95% CI)	OR (95% CI)	P-value	% (95% CI)
**Glycemic index [1]**																	
By food item																	
Low	42.1 (27.4-58.4)	9.7 (6.3-14.6)	1.00 (-)		54.2 (34.1-72.9)	9.5 (6.3-14.2)	1.00 (-)		43.3 (26.8-61.5)	10.8 (7.2-15.7)	1.00 (-)		46.9 (30.3-64.2)	9.0 (5.8-13.7)	1.00 (-)		13.9 (10.1-18.9)
Medium	28.9 (16.6-45.4)	77.7 (71.4-82.9)	0.08 (0.03-0.19)	<0.001	20.8 (8.7-42.0)	76.4 (70.3-81.5)	0.05 (0.02-0.15)	<0.001	20.0 (9.1-38.4)	78.0 (71.9-83.1)	0.06 (0.02-0.17)	<0.001	18.8 (8.5-36.4)	78.8 (72.7-83.8)	0.05 (0.02-0.13)	<0.001	70.9 (64.8-76.3)
High	28.9 (16.6-45.4)	12.6 (8.7-17.9)	0.48 (0.18-1.26)	0.136	25.0 (11.4-46.3)	14.1 (10.1-19.4)	0.31 (0.10-0.95)	0.041	36.7 (21.3-55.3)	12.1 (8.4-17.3)	0.68 (0.25-1.83)	0.45	34.4 (19.9-52.5)	12.3 (8.5-17.4)	0.54 (0.20-1.42)	0.211	15.2 (11.2-20.3)
Weighted by frequency of consumption																	
Low	35.7 (11.5-70.5)	10.1 (2.7-31.3)	1.00 (-)		65.8 (30.5-89.3)	10.1 (2.8-30.6)	1.00 (-)		36.3 (9.8-75.0)	10.2 (2.8-31.2)	1.00 (-)		38.3 (11.1-75.5)	10.1 (2.7-31.2)	1.00 (-)		11.2 (3.4-31.1)
Medium	15.5 (4.5-41.7)	51.6 (17.7-84.1)	0.08 (0.01-0.64)	0.017	11.5 (3.0-35.2)	50.9 (17.9-83.1)	0.03 (0.00-0.28)	0.002	6.8 (1.8-22.7)	51.7 (17.9-84.1)	0.04 (0.00-0.30)	0.002	6.6 (1.8-2.2)	51.8 (17.9-84.1)	0.03 (0.00-0.26)	0.001	50.1 (18.1-82.0)
High	48.7 (16.9-81.6)	38.3 (9.0-79.5)	0.34 (0.03-4.90)	0.441	22.7 (5.5-59.6)	39.0 (9.9-79.0)	0.09 (0.01-1.18)	0.066	56.8 (20.1-87.3)	38.0 (9.0-79.3)	0.42 (0.03-6.01)	0.522	55.1 (19.5-86.1)	38.1 (9.0-79.3)	0.38 (0.03-5.31)	0.473	38.7 (10.0-78.3)
**Glycemic load [2]**																	
By food item																	
Low	44.7 (29.7-60.8)	16.0 (11.6-21.7)	1.00 (-)		66.7 (45.6-82.7)	15.5 (11.2-20.9)	1.00 (-)		53.3 (35.4-70.4)	15.9 (11.5-21.5)	1.00 (-)		53.1 (35.8-69.8)	15.6 (11.3-21.1)	1.00 (-)		20.5 (15.9-26.1)
Medium	39.5 (25.2-55.8)	44.7 (38.0-52.0)	0.32 (0.14-0.70)	0.005	25.0 (11.4-46.3)	45.9 (39.4-52.6)	0.13 (0.05-0.35)	<0.001	33.3 (18.7-52.1)	45.3 (38.7-52.1)	0.22 (0.09-0.53)	0.001	31.3 (17.5-49.4)	45.8 (39.1-52.5)	0.20 (0.08-0.48)	<0.001	43.9 (37.7-50.2)
High	15.8 (7.2-31.3)	39.3 (32.8-46.2)	0.14 (0.05-0.40)	<0.001	8.3 (2.0-28.7)	38.6 (32.4-45.3)	0.05 (0.01-0.23)	<0.001	13.3 (5.0-31.1)	38.8 (32.4-45.5)	0.10 (0.03-0.33)	<0.001	15.6 (6.5-32.9)	38.7 32.3-45.5	0.12 (0.04-0.35)	<0.001	35.7 (29.9-41.9)
Weighted by frequency of consumption																	
Low	33.6	3.9	1.00		70.7	3.8	1.00		39.0	3.9	1.00		38.0	3.9	1.00		5.2

	(10.4-68.7)	(1.3-11.0)	(-)		(35.1-91.5)	(1.3-10.6)	(-)		(11.0-76.9)	(1.3-10.9)	(-)		(11.0-75.3)	(1.3-10.9)	(-)		(2.0-13.0)
Medium	55.4 (22.3-84.3)	51.8 (17.8-84.2)	0.12 (0.02-0.90)	0.039	16.7 (4.4-46.9)	52.7 (18.7-84.4)	0.02 (0.00-0.12)	<0.001	51.4 (16.0-85.4)	52 (18.0-84.2)	0.1 (0.01-0.85)	0.035	49.8 (15.5-84.3)	52.1 (18.0-84.3)	0.1 (0.01-0.84)	0.034	52 (18.9-83.4)
High	11 (2.8-34.3)	44.3 (13.0-80.8)	0.03 (0.00-0.28)	0.002	12.6 (1.7-53.8)	43.5 (12.9-80.0)	0.02 (0.00-0.24)	0.003	9.6 (1.9-37.4)	44.1 (13.0-80.6)	0.02 (0.00-0.25)	0.002	12.2 (2.9-38.9)	44 (13.0-80.6)	0.03 (0.00-0.28)	0.002	42.8 (12.8-79.3)

CI, confidence interval.
[1] Low GI ≤ 55, medium GI 56-69, high GI ≥70.
[2] Low GL ≤10, medium GL 11-19, high GL ≥20.
[3] Metrics were applied per 10g of total carbohydrate: [1] ≥1g fiber (10:1), [2] ≥1g fiber and <1g free sugars (10:1:1), [3] ≥1g fiber and <2g free sugars (10:1:2); and [4] ≥1g fiber and, per 1 g of fiber, < 2g free sugars (10:1|1:2).
[4] Yes, foods that met the respective carbohydrate metric.
[5] No, foods that did not meet the respective carbo-hydrate metric.

When weighted by the population frequency of consumption, a higher proportion of grain foods had higher GI when compared to the non-weighted analysis (38.7% vs. 15.2%, respectively) indicating that food items with higher GI were more frequently consumed (Supplementary Table 4). All carbohydrate metrics identified food items more likely to be low GI than medium GI (P-value<0.05). More than 1 in each 3 foods meeting the metrics were low GI (ranging from 35.7% to 65.8% across metrics), compared to only 1 in 10 foods not meeting the metrics (ranging from 10.1% to 10.2%). However, approximately half of foods meeting the metrics were high GI (ranging from 22.7% to 56.8%), compared with 4 in 10 foods not meeting the metrics (ranging from 38.0% to 39.0%) (Table S5).

According to categories of GL, higher proportion of grain foods had higher GL when the weighted analysis was compared to the non-weighted analysis (42.8% vs. 35.7%, respectively) (Table S5). All carbohydrate metrics identified food items more likely to be low GL than medium or high GL (P-value<0.05), and more than 1 in each 3 foods meeting the metrics were low GI (ranging from 33.6% to 70.7% across metrics), compared to less than 1 in 10 foods not meeting the metrics (ranging from 3.8% to 3.9%).

Table S6. Comparison of consumed grain foods meeting at least one criterion of the front-of-pack warning labels according to carbohydrate metrics, 2015 Health Survey of Sao Paulo (n=244).

Carbohydrate metrics [1]	Front-of-pack warning labels: ANVISA [2]		Chile 1rst stage		Chile 3rd stage		PAHO	
	% (95%CI)	P-value	% (95%CI)	P-value	% (95%CI)	P-value	% (95%CI)	P-value
10:1								
No [3]	70.4 (63.7-76.3)	<0.001	70.4 (63.7-76.3)	0.001	87.9 (82.6-91.7)	0.001	88.3 (83.2-92.1)	0.002
Yes [4]	26.3 (14.6-42.7)		36.8 (23.0-53.3)		65.8 (49.3-79.2)		68.4 (51.9-81.3)	
10:1:1								
No	69.1 (62.6-74.9)	<0.001	69.1 (62.6-74.9)	<0.001	88.2 (83.2-91.8)	<0.001	89.1(84.2-92.6)	<0.001
Yes	12.5 (4.0-33.1)		29.2 (14.3-50.4)		50.0 (30.5-69.5)		50.0 (30.5-69.5)	
10:1:2								
No	70.6 (64.1-76.3)	<0.001	70.1 (63.6-75.9)	<0.001	88.3 (83.2-92.0)	<0.001	88.8 (83.8-92.4)	<0.001
Yes	13.3 (5.0-31.1)		30.0 (16.2-48.8)		56.7 (38.5-73.2)		60.0 (41.6-76.0)	
10:1|1:2								
No	70.3 (63.7-76.1)	<0.001	69.8 (63.2-75.7)	<0.001	88.2 (83.1-91.9)	<0.001	88.7 (83.6-92.3)	<0.001
Yes	18.8 (8.5-36.4)		34.4 (19.9-52.5)		59.4 (41.6-75.0)		62.5 (44.5-77.6)	
Overall	63.5 (57.3-69.4)		65.2 (58.9-70.9)		84.4 (79.3-88.5)		85.2 (80.2-89.2)	
**Weighted by frequency of consumption [5]**								
10:1								
No	40.7 (13.1-75.8)	0.28	21.8 (8.3-46.0)	0.90	46.8 (15.8-80.4)	0.40	99.5 (97.5-99.9)	0.37
Yes	18.5 (5.7-46.1)		23.6 (7.8-53.2)		68.6 (32.6-90.8)		99.0 (91.6-99.9)	
10:1:1								
No	40.4 (13.3-74.9)	0.14	21.8 (8.5-45.4)	0.86	47.9 (16.8-80.8)	0.70	48.0 (9.17- 80.8)	0.67
Yes	10.7 (1.97-41.9)		24.9 (6.5-61.0)		37.7 (12.1-72.7)		36.9 (11.8-71.9)	
10:1:2								
No	41.1 (13.4-75.8)	0.05	22.1 (8.5-46.4)	0.59	47.1 (16.1-80.5)	0.56	47.1 (16.1-80.5)	0.52
Yes	6.4 (1.2-27.4)		14.6 (3.7-43.1)		63.4 (24.8-90.1)		65.2 (26.0-90.9)	
10:1|1:2								
No	41.0 (13.3-75.8)	0.07	22.0 (8.4-46.3)	0.73	47.0 (16.0-80.5)	0.53	47.0 (16.0-80.5)	0.49
Yes	9.3 (2.3-30.7)		17.2 (4.9-45.4)		64.5 (26.4-90.2)		66.2 (27.6-91.0)	
Overall	39.8 (13.4-73.9)		21.9 (8.7-45.0)		47.7 (17.1-80.1)		47.7 (9.2-80.2)	

CI, confidence interval.

[1] Metrics were applied per 10g of total carbohydrate: 1) ≥1g fiber (10:1), 2) ≥1g fiber and <1g free sugars (10:1:1), 3) ≥1g fiber and <2g free sugars (10:1:2); and 4) ≥1g fiber and, per 1 g of fiber, < 2g free sugars (10:1|1:2).

[2] The National Health Surveillance Agency (*Agência Nacional de Vigilância Sanitária* - Anvisa) front-of-packing warning label tags foods with saturated fat ≥6 g/100 g, added sugar ≥15 g/100 g, or sodium ≥600 mg/100 g. Grains and flours without sugar, saturated fat or sodium added to the product were not eligible to receive the warning label (n=20). The 1st stage of Chilean warning label tagged foods with energy ≥350 kcal/100 g, saturated fat ≥6 g/100 g, total sugar ≥22.5 g/100 g, or sodium ≥800 mg/100 g. In the 3rd stage, foods with energy ≥275 kcal/100 g, saturated fat ≥4 g/100 g, total sugar ≥10 g/100 g, sodium ≥40 mg/100 g are tagged. Foods without sugar, sodium or fat added to the product were not eligible to receive the Chilean warning label (n=27). The Pan American Health Organization (PAHO) warning label tags processed and ultraprocessed foods with total fat ≥30% food total energy (FTE), saturated fat ≥10% FTE, trans fat ≥1% FTE, free sugar ≥10% FTE, sodium ≥1mg/1 kcal, or the presence of non-nutritive sweetener (n=216).

[3] No, foods that did not meet the respective carbo-hydrate metric.

[4] Yes, foods that met the respective carbo-hydrate metric.

[5] Products were weighted by their actual reported consumption levels (products consumed more frequently received larger weight).

The proportion of grain products receiving at least one warning label was lower when the frequency of consumption was considered for all systems considered (Table S6), indicating that food items with at least one warning label were less frequently consumed. Results weighted by the population frequency of consumption were not significant when the foods meeting at least one criterion of the front-of-pack warning labels were compared according to meeting or not meeting the carbohydrate metrics (Table S6).

Table S7. Contents of critical nutrients in nutrient profiling models according to carbohydrate quality metrics, 2015 Health Survey of Sao Paulo.

Critical nutrients [1]	10:1 [2]						10: 1:1					
	Yes [3]		No [4]		Diff. (SE)[5]	P-value	Yes		No		Diff. (SE)	P-value
	μ (SE)	med (IQR)	μ (SE)	med (IQR)			μ (SE)	med (IQR)	μ (SE)	med (IQR)		
Energy (kcal)	315.4 (14.5)	339.6 (150.1)	337.5 (7.1)	349.2 (140.6)	-22.2 (16.0)	0.17	313.7 (18.9)	340.7 (138.1)	336.3 (6.8)	348.4 (143.2)	-22.6 (19.7)	0.25
Total sugar (g)	8.4 (1.8)	3.9 (14.3)	19.8 (1.1)	20.9 (29.1)	-11.4 (2.1)	<0.001	2.7 (0.9)	0.8 (3.3)	19.7 (1.0)	20.3 (28.2)	-17.0 (1.3)	<0.001
Free sugar (g)	5.4 (1.3)	0.9 (10.0)	17.2 (1.1)	17.1 (28.0)	-11.8 (1.7)	<0.001	0.71 (0.3)	0.1 (0.4)	17.0 (1.0)	15.6 (26.7)	-16.3 (1.1)	<0.001
Added sugar (g)	5.4 (1.3)	0.9 (10.0)	17.1 (1.1)	16.9 (28.0)	-11.7 (1.7)	<0.001	0.71 (0.3)	0.1 (0.4)	16.9 (1.0)	15.6 (26.7)	-16.2 (1.1)	<0.001
Total fat (g)	8.0 (1.2)	6.2 (10.2)	11.4 (0.5)	10.2 (12.5)	-3.4 (1.3)	0.01	6.9 (1.4)	5.2 (7.6)	11.3 (0.5)	10.4 (12.6)	-4.4 (1.5)	0.004
Saturated fat (g)	2.2 (0.6)	1.1 (1.7)	4.3 (0.3)	3.3 (5.3)	-2.1 (0.7)	0.001	1.8 (0.7)	0.8 (1.2)	4.3 (0.3)	3.2 (5.3)	-2.5 (0.7)	0.001
Trans fat (g)	0.2 (0.1)	0.1 (0.2)	0.8 (0.1)	0.2 (0.7)	-0.6 (0.1)	<0.001	0.1 (0.1)	0.1 (0.1)	0.8 (0.1)	0.2 (0.6)	-0.7 (0.1)	<0.001
Sodium (mg)	263.7 (43.4)	176.0 (456.4)	277.7 (17.7)	225.5 (383.0)	-14.0 (46.5)	0.76	274.8 (61.2)	192.0 (479.9)	275.6 (16.9)	219.4 (390.6)	-0.8 (62.5)	0.99

Critical nutrients [a]	10:1:2						10:1|1:2					
	Yes		No		Diff. (SE)	P-value	Yes		No		Diff. (SE)	P-value
	μ (SE)	med (IQR)	μ (SE)	med (IQR)			μ (SE)	med (IQR)	μ (SE)	med (IQR)		
Energy (kcal)	306.5 (16.2)	335.4 (143.0)	338.0 (6.7)	349.2 (144.3)	-31.5 (17.4)	0.07	313.6 (15.9)	340.7 (144.7)	337.2 (6.9)	348.4 (141.8)	-23.6 (17.2)	0.173
Total sugar (g)	4.7 (1.5)	1.5 (4.4)	20.0 (1.1)	20.8 (28.6)	-15.3 (1.8)	<0.001	5.6 (1.6)	2.6 (4.7)	19.9 (1.1)	20.9 (28.8)	-14.3 (1.9)	<0.001
Free sugar (g)	2.0 (0.6)	0.1 (4.0)	17.2 (1.0)	16.3 (27.3)	-15.3 (1.2)	<0.001	2.8 (0.8)	0.0 (4.2)	17.3 (1.0)	16.5 (27.5)	-14.3 (1.3)	<0.001
Added sugar (g)	2.0 (0.6)	0.1 (4.0)	17.2 (1.0)	16.3 (27.3)	-15.2 (1.2)	<0.001	2.8 (0.8)	0.0 (4.2)	17.2 (1.0)	16.4 (27.5)	-14.3 (1.3)	<0.001
Total fat (g)	6.3 (1.2)	3.1 (7.3)	11.5 (0.5)	10.7 (12.3)	-5.1 (1.3)	<0.001	6.7 (1.1)	3.9 (8.8)	11.5 (0.5)	10.5 (12.3)	-4.8 (1.3)	<0.001
Saturated fat (g)	1.6 (0.6)	0.7 (1.2)	4.4 (0.3)	3.3 (5.2)	-2.8 (0.7)	<0.001	1.6 (0.5)	0.7 (1.5)	4.4 (0.3)	3.3 (5.2)	-2.8 (0.6)	<0.001
Trans fat (g)	0.1 (0.1)	0.1 (0.2)	0.8 (0.1)	0.2 (0.7)	-0.7 (0.1)	<0.001	0.1 (0.0)	0.0 (0.1)	0.8 (0.1)	0.2 (0.7)	-0.7 (0.1)	<0.001
Sodium (mg)	301.3 (52.7)	262.0 (472.3)	271.9 (17.2)	210.4 (376.0)	29.4 (54.8)	0.59	291.5 (49.8)	224.0 (466.1)	273.1 (17.3)	217.2 (376.2)	18.5 (52.2)	0.724

μ, mean; SE, standard error; med, median; IQR, interquartile range; Diff., difference.

[1] Sweeteners were excluded from the analyses due to lack of information regarding amount added to products available in Brazil.

[2] Metrics were applied per 10g of total carbohydrate: [1] ≥1g fiber (10:1), [2] ≥1g fiber and <1g free sugars (10:1:1), [3] ≥1g fiber and <2g free sugars (10:1:2); and [4] ≥1g fiber and, per 1 g of fiber, < 2g free sugars (10:1|1:2).

[3] Yes, foods that met the respective carbo-hydrate metric.

[4] No, foods that did not meet the respective carbo-hydrate metric.

[5] β coefficient for linear regression model with robust variance.

Figure S1. Flowchart of study participants, 2015 Health Survey of Sao Paulo.

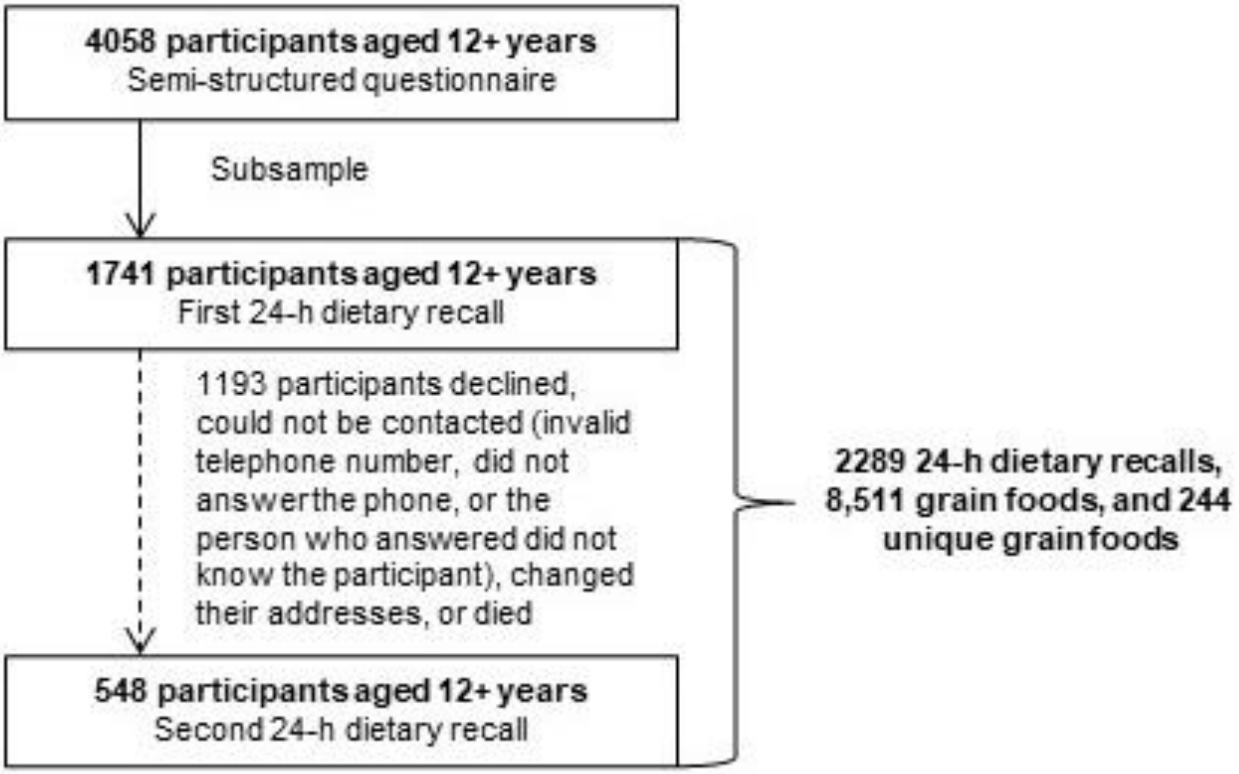

Supplement: Supplementary file 1 [file foods-13-01299-s001.zip › foods-2940948-supplementary.pdf]
